# High Temperature Flow Behavior of Ultra-Strong Nanoporous Au assessed by Spherical Nanoindentation

**DOI:** 10.3390/nano8060366

**Published:** 2018-05-24

**Authors:** Alexander Leitner, Verena Maier-Kiener, Daniel Kiener

**Affiliations:** 1 Department Materials Physics, Montanuniversität Leoben, Jahnstraße 12, A-8700 Leoben, Austria; alexander.leitner@unileoben.ac.at; 2 Department Physical Metallurgy and Materials Testing, Montanuniversität Leoben, Roseggerstraße 12, A-8700 Leoben, Austria; verena.maier kiener@unileoben.ac.at

**Keywords:** spherical nanoindentation, nanoporous Au, high temperature testing, mechanical properties

## Abstract

Nanoporous metals have attracted attention in various research fields in the past years since their unique microstructures make them favorable for catalytic, sensory or microelectronic applications. Moreover, the refinement of the ligaments down to the nanoscale leads to an exceptionally high strength. To guarantee a smooth implementation of nanoporous metals into modern devices their thermo-mechanical behavior must be properly understood. Within this study the mechanical flow properties of nanoporous Au were investigated at elevated temperatures up to 300 °C. In contrast to the conventional synthesis by dealloying of AuAg precursors, the present foam was fabricated via severe plastic deformation of an AuFe nanocomposite and subsequent selective etching of iron, resulting in Au ligaments consisting of nanocrystalline grains, while remaining Fe impurities excessively stabilize the microstructure. A recently developed spherical nanoindentation protocol was used to extract the stress-strain curves of nanoporous Au. A tremendous increase of yield strength due to ligament and grain refinement was observed, which is largely maintained at high temperatures. Reviewing literature will evidence that the combined nanocrystalline and nanoporous structure leads to remarkable mechanical properties. Furthermore, comparison to a previous Berkovich nanoindentation study outlines the conformity of different indentation techniques.

## 1. Introduction

Nanoporous (NP) gold has been in the focus of interest in the material science community for decades as the reduction of ligament width has turned out to enhance the strength of the material in an extraordinary manner [1,2,3,4,5,6,7,8]. Owed to this fact, also highly porous and consequently light weight structures can endure considerable loads. Dealloying of AuAg precursors is the dominating fabrication process of NP Au [9,10,11]. However, this is accompanied by the drawback that single grains extend over a high number of ligaments which is detrimental for the strength of the material [9,10,11]. The high purity of these foams is beneficial for the investigation of deformation mechanisms because other tailoring mechanisms become negligible but thereby the structures have a strong tendency to coarsen even at intermediate homologue temperatures (*T_hom_* > 0.3) [11,12,13,14]. An alternative fabrication route via severe plastic deformation (SPD) of immiscible metals and subsequent selective etching can kill two birds with one stone. On the one hand, the grain size can be tremendously reduced down to length scales smaller than the average ligament width [15]. On the other hand, small amounts of the second phase remain in the material due to supersaturation after mechanical mixing and stabilize the microstructure up to 400 °C [15,16,17]. For the conventional synthesis of NP Au, the difference of nobility of the solid solution precursor constituents and apparent surface diffusion are decisive, while an electric potential must be applied to stimulate the process resulting in an essentially single crystalline structure. The etching approach used within this study is based on the selective attack of the used acid on the base metal (Fe) of a two-phase precursor, hence no material diffusion mechanisms are involved and a nanocrystalline structure is preserved. The deformation behavior at room temperature (RT) and high temperatures (HT) has been investigated in a prior study utilizing Berkovich nanoindentation experiments for the extraction of elastic, plastic and time-dependent properties [15]. However, as pyramidal tips introduce a self-similar deformation at rather high representative strains ($\varepsilon_{i}\approx$ 7.2% for the Berkovich geometry [18]) information about the onset of plasticity and the work hardening is lost. Also, strain-dependent densification effects cannot be identified. Using a spherical indentation approach allows to continuously increase the strain during indentation and opens the possibility to extract the entire flow curve with a single indent if dynamic measurement techniques such as continuous stiffness measurement (CSM) are applied [19,20,21]. Subject of this study is to investigate the flow behavior of NP Au up to 300 °C to evaluate the role of temperature concerning the mechanical properties of an ultra-strong Au foam.

## 2. Materials and Methods.

### 2.1. Precursor Synthesis

**Figure 1:**
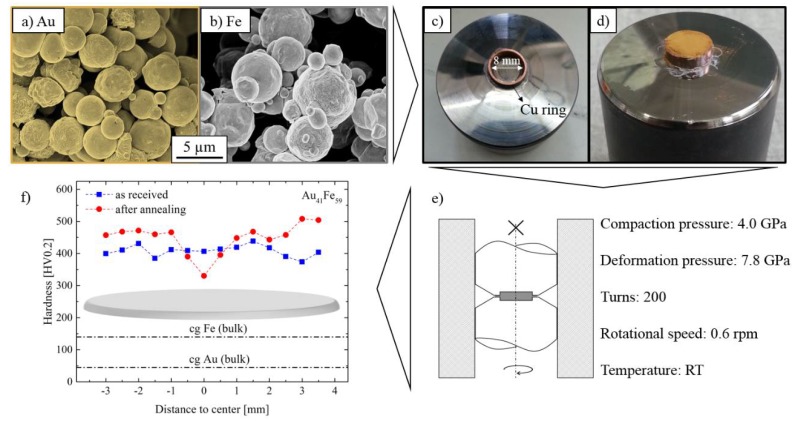
Scanning electron microscope (SEM) images of (**a**) Au powder (image colored ex post) and (**b**) Fe powder used as a base material for the composite precursor; (**c**) Lower anvil of the HPT setup with affixed Cu ring used (**d**) as powder container; (**e**) Scheme of HPT with used compaction and deformation parameters; (**f**) Microhardness measurements of the deformed Au_41_Fe_59_ composites show a softening in the center and hardening closer to the edge upon annealing.

Au and Fe were selected to fabricate the precursor due to their extended miscibility gap and low solubility in each other at ambient temperatures, a sine quo non to achieve a fairly pure Au structure. The Au powder (Alpha Aesar, Ward Hill, MA, USA purity 3N6, 1250 Mesh) and Fe powder (Merck, Darmstadt, Germany, purity 3N, 1250 Mesh) particles have a spherical character with low affinity to agglomeration (see Figure 1a,b), which is required to generate a homogeneous batch in the subsequent multiaxial mixing process. To achieve the desired porosity of 50%, the powders have to be mixed 50/50 vol % resulting in a ratio of 41 at % Au to 59 at % Fe (Au_41_Fe_59_). Note that both phases must exhibit a continuous network to facilitate the following etching process which accordingly limits the obtainable porosities. The applied SPD technique must serve two purposes: On the one hand, the mixed powders must be compressed to a blank part. On the other hand, it must further refine the sample’s microstructure to reduce the grain size down to the nanoscale and homogenize the sample. High pressure torsion (HPT) fulfills these requirements and allows a straight-forward fabrication process [16,22,23]. A thin-walled copper ring with an inner diameter of 8 mm is glued to the lower anvil of the HPT device to contain the powder mix (Figure 1c,d). In the successive step, the pressure is slowly increased up to 4.0 GPa when the upper anvil approaches the ring. This ensures a lasting compaction of the powder particles. After unloading, the Cu ring is removed from the outer edge of the created disk to prevent impurifications of the generated AuFe composite by Cu. Thereafter, the actual severe plastic deformation step is performed by reloading the AuFe disk with a pressure of 7.8 GPa and by rotating the anvils against each other with a rotational speed of 0.6 rpm until 200 turns are reached (Figure 1e). This corresponds to an equivalent strain of around 4500 at the outer radius of 4 mm. For a closer mechanical and microstructural analysis, the samples are mechanically ground and polished. Despite the immiscibility in the thermodynamic equilibrium the extreme strains reached during HPT cause strong supersaturation effects [16,17]. Annealing of the precursors at 300 °C for 1 h will counteract this phenomenon and promote the segregation of Au and Fe, respectively. Figure 1f shows the microhardness in dependence of disk radius *r* for as received (aR) and annealed Au_41_Fe_59_. At regions more than 1 mm off the center a distinct hardening due to annealing is observed from approximately 400 HV to 450 HV. This has been correlated to grain-boundary (GB) relaxation effects which reduce internal stresses and impede the generation of dislocations necessary to bear the deformation by the indenter [24,25]. Since the shear strain induced by HPT is dependent on the radius, the material close to the center will experience less deformation and thereby a coarser microstructure remains, related to lower hardness values. Thus, mentioned mechanisms are not dominant anymore and the reduction of the dislocation density leads to lower hardness values compared to the aR sample (400 HV to 325 HV) in the disk center as expected from the classical Taylor hardening model [26]. Minor deviations from a constant hardness level might result from emerging shear bands which form during the HPT process [27].

### 2.2. Selective Etching Process

Fe can be removed by applying 5 wt % HCl for 24 h at a temperature of 55 °C. These parameters were evaluated from the Pourbaix diagram of Fe [28] and also used to obtain NP Cu previously [29]. As Au is stable under these conditions a one-phase network remains. Further hardening by annealing of the present foam has been observed in a recent study [15], hence the fabricated foam was annealed at 300 °C for 1 h to avoid further hardening during HT testing.

### 2.3. Mechanical Characterization by Spherical Nanoindentation

Micromechanical testing techniques are a necessity to examine modern materials. While uniaxial micro-compression and micro-tension tests allow a straightforward data analysis, the sample preparation is fairly elaborate. In addition, it is an ambitious balancing act to achieve a stiff and well aligned testing setup [30,31]. Spherical indentation is an alternative testing method to gain a material’s flow curve where the sample preparation time is vastly reduced as solely a well-polished surface is needed [18,19,20,21,32]. The non-self-similar tip character induces a continuously increasing strain with increasing displacement, where the corresponding hardness can be converted into a representative indentation stress by a strain and material dependent constraint factor C* [18,33,34,35]. Previously, it was demonstrated that for materials with refined microstructure and negligible indentation size effect the obtained indentation flow curves are in good agreement with data from purely uniaxial tests [21]. However, the choice of the value for C*, which is related to the morphology of the plastic zone underneath the indenter tip, demands some more considerations for foams where the material is only marginally constrained. By the induced deformation the local plastic zone within the fine ligaments will almost instantly reach the free surface. This rather leads to states referred to as fully developed plastic zones, for which C* has been ascertained to be constant in the range of 2.5 to 3 [18,35]. One should not misleadingly use C* ≈ 1 since that value refers to a purely elastic deformation and not simply to an unconstrained deformation as it is the case for NP metals. Considering the Poisson ratio of the present foam of *ν* = 0.2 (according to Luehrs et al. [36]) an estimated value close to C* = 2.5 is reasonable and supported by a variety of previous studies [37,38,39,40,41]. Further details of the used spherical nanoindentation approach and the conducted analysis are given in [21].

To assess the strength of the foam ligaments themselves, the estimation suggested by Gibson and Ashby can be used [37]:

(1) $$\sigma_{y,lig}=\frac{\sigma_{y}}{A\cdot {\left(1-p\right)}^{B}}$$

where $\sigma_{y,lig}$ is the yield strength of the ligaments, $\sigma_{y}$ is the global yield strength of the foam, *p* is the porosity and *A* = 0.5 and *B* = 1.5 are constants referring to the open-cellular structure. The yield strength we refer to within this study accords to a plastic strain of 0.2%. The Young’s modulus *E* of the tested sample is determined using the conventional analysis of Oliver and Pharr, which is based on Sneddon’s solution of the indentation relationship [42,43]. Again, using the concept of Gibson and Ashby allows to estimate the Young’s modulus of the ligaments *E_lig_* [37]:

(2) $$E_{lig}=\frac{E}{C\cdot {\left(1-p\right)}^{D}}$$

where *C* = 1 and *D* = 2, attributed to a regular open-cell geometry.

At least six indentation tests were performed for each temperature and different microstructure of the investigated foam using a Nanoindenter G200 (KLA-Tencor, Livermore, CA, USA). The thermal drift was measured in a post-test hold segment at 10% of the maximum load for each indent. Tests exceeding a drift value of 0.3 nm/s were not considered for the analysis. The strain-rate for spherical indentation tips was set to ${\dot{\varepsilon }}_{i}$ = 1 × 10^−3^ s^−1^. Strain-rate controlled testing is essential since nanoporous Au features a significant rate-dependency originating from dislocation-interface interactions due to the high fraction of free surfaces but also grain boundaries [15].

For high temperature testing a laser-based heating system (Surface Tec, Hückelhoven, Germany) was utilized were tip and sample are independently heated by a laser beam guided through a glass-fiber to the region of interest. Details of the used HT-setup can be found, for example, in a review of Wheeler et al. [44]. RT and HT tip have specified radii of 20 µm and are made from diamond (*E* = 1140 GPa, *ν* = 0.07, Synton MPD LTD, Nidau, Switzerland) and sapphire (*E* = 440 GPa, *ν* = 0.28, Synton MPD LTD, Switzerland & Surface Tec, Hückelhoven, Germany), respectively. Note that smaller tip radii could lead to falsified results since recently NP Au was found to feature a pronounced indentation size effect [44,45]. Beside testing at RT, experiments were also conducted at temperatures of 100 °C, 200 °C and 300 °C. After cooling down the sample to RT, reference indentation tests were performed to check whether the initial properties were modified due to the HT exposure.

## 3. Results and Discussion

### 3.1. Characterization of Nanoporous Au

**Figure 2:**
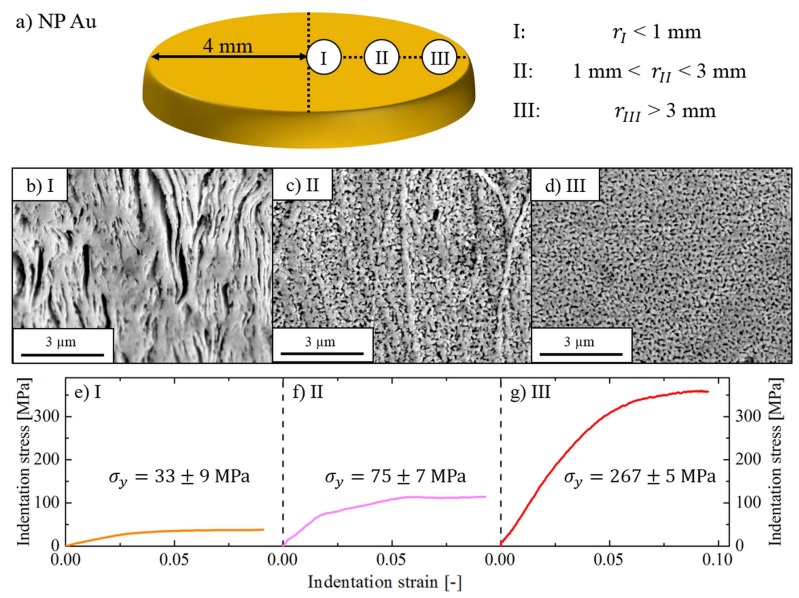
(**a**) Schematic overview of the HPT disk with investigated areas; (**b**–**d**) SEM images of microstructural features of NP Au in dependence of the disk radius; (**e**–**g**) Corresponding spherical indentation stress-strain curves of the selected regions.

Due to the gradual deformation by HPT no uniform microstructure is expected [22,23]. Therefore, the impact of the sample’s inhomogeneity was examined. Figure 2a gives an overview of the investigated positions of the obtained NP Au disk. The microstructure strongly varies depending on the radial position, even though the global porosity is constantly 0.5. In the center (*r_I_* < 1 mm, Figure 2b) no uniform structure was observed which complicates nanoindentation tests as the observed properties are mainly dependent on the local indentation-site topography [46]. Spherical indentation performed in region *r_I_* revealed $\sigma_{y}$ = 33 ± 8 MPa, corresponding to a ligament yield strength of $\sigma_{y,lig}$ = 311 ± 85 MPa (Figure 2e). Moving towards the disk edge (1 mm < *r_II_* < 3 mm, Figure 2c) the foam becomes more homogenous and the strength significantly increases to values up to $\sigma_{y}$ = 75 ± 7 MPa or $\sigma_{y,lig}$ = 707 ± 69 MPa (Figure 2f). Still, the indentation flow curve shows a rather discontinuous profile which might arise from the remaining variations within the probed microstructure. Naturally, the regions where the material endured the highest strain near the edge (*r_III_* > 3 mm, Figure 2d) feature the most homogenous structure in this region. The stress-strain curve becomes smooth and outstanding strength values of $\sigma_{y}$ = 267 ± 5 MPa and $\sigma_{y,lig}$ = 2517 ± 45 MPa are reached. The relative standard deviation decreases drastically from 27% in the center to less than 2% close to the edge, thereby confirming the high homogeneity of the foam (Figure 2g). Previous FIB cross-sectional analysis further substantiates a uniform structure. Compared to the disk center, the strength increases by a factor of 8 even though *p* remains constant. This quantitatively confirms the enormous impact of structure refinement on the material’s strength by grain boundaries and free surfaces [3,7,47,48,49].

Due to the exceptional properties of the outer region *r_III_* this area is investigated in depth at HT. In detail, the average ligament diameter was evaluated by gauging at least 30 individual ligaments at their narrowest point, using high magnification scanning electron microscope (SEM) images. The analysis results in a value of approximately 100 nm. The grain size, on the other hand, could be obtained from electron backscatter diffraction (EBSD, EDAX Inc., Mahwah, NJ, USA), using a threshold angle of 15° to discriminate low- and high-angle grain boundaries. A mean grain size of around 70 nm was determined for the fabricated NP Au, assuming spherical grains. Consequently, ligaments predominantly consist of several grains while single crystalline ligament cross-sections become improbable, considering the grain dimension and shape. Also, no significant texture effects due to HPT could be identified. Further details concerning the thermal stability of the microstructure are given in [15].

### 3.2. High Temperature Nanoindentation

**Figure 3:**
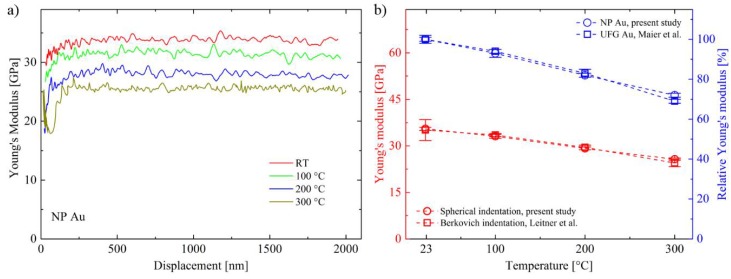
(**a**) Young’s modulus profiles of spherical HT nanoindentation tests up to 300 °C. The absence of significant gradients indicates reliable measurements at all temperatures; (**b**) The linear decrease of *E* over temperature is in excellent agreement with Berkovich measurements of a previous study (red data) [15]. Furthermore, the relative decrease of *E* over *T,* related to the room temperature value of each material, coincides well with data from ultra-fine grained Au (blue data) [57].

Spherical indentation studies at non-ambient temperatures are seldom so far. Advancing the technique towards flow curve extraction might further increase the interest as HT properties are important parameters for metal forming processes or components operating at elevated temperatures. One challenge for the sensible technique of nanoindentation is to check for the measurement reliability, especially at HT where sample/tip interactions, thermal drift or sample fixation are demanding [50,51,52,53]. Using a dynamic indentation technique such as CSM enables to record the profile of the Young’s modulus continuously over displacement which should feature a horizontal profile over penetration depth for isotropic materials, as it is the case for the present NP Au. Deviations from a constant value could originate from pile-up or sink-in effects, but also and more importantly from an incorrect machine compliance or erroneous tip calibrations [42,54,55,56]. If this is the case, the obtained data must be critically analyzed and existing models to consider these effects should be applied. However, as to see from Figure 3, the data obtained within this study fulfill the condition of constant *E* profiles and thereby indicate a high reliability of the used method at all temperature conditions. Apparently, also occurring densification underneath the indenter tip does not decisively manifest in the recorded data, as the elastic field spans over a manifold of the plastic zone. Figure 3b shows average values of the Young’s modulus over temperature, determined at displacements between 500 and 2000 nm. The expected slight monotonic decrease of *E* results from thermally induced weakening of atomic bonds (red open circles). These values are in excellent accordance with measurements of a previous study performed with a Berkovich tip (red open squares) and confirms that despite different tip characters the same elastic properties are obtained using the relation of Sneddon [15,21,43]. Admittedly, employing Equation (2) results in *E* values up to 140 GPa, which is well above expectations from Au bulk values of max. 97 GPa, already considering the remaining Fe [15,57]. Certainly, the Gibson and Ashby model is under debate for NP materials as it was derived for regular open-cell structures of low density foams [7,58,59,60]. Experiments and simulations conclude that real NP metals behave differently and are more sensitive to deviating porosities and impurities than expected from the classical models [37,58,59]. Comparison to HT data of ultra-fine grained Au, made from the same base material, shows an equivalent profile of *E* over *T* if the Young’s modulus is normalized in regard to the RT value (Figure 3b, blue symbols). This means that the high fraction of free surfaces seems not to further influence the intrinsic elastic response of the material.

**Figure 4:**
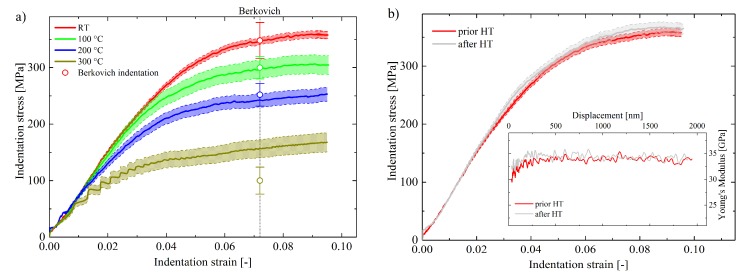
(**a**) Indentation flow curves with according deviations of NP Au at room and elevated temperatures. Except the data measured at 300 °C Berkovich indentation tests of previous studies coincide well with spherical measurements; (**b**) Comparison of measurements conducted prior and after HT exposure does not reveal any changes of the mechanical properties. The elastic modulus (inset) remains unaffected by re-annealing.

The nanoindentation flow curves displayed in Figure 4a demonstrate a weakening of NP Au with increasing temperature. Thereby, $\sigma_{y}$ decreases gradually from 267 ± 5 MPa at RT down to 119 ± 12 MPa at 300 °C. In general, work hardening is observable for all curves which mainly originates from a non-self-similar densification of the foam contributing to apparent higher stress values at higher strains [5,40,61]. The character of the work-hardening behavior is slightly temperature-affected and transforms from a rather parabolic characteristic at RT to a linear hardening behavior at higher temperatures. Even though the yield strength at 300 °C is clearly reduced compared to the RT value, back calculation of the ligament strength assessed by Equation (1) still results in extraordinary high values of $\sigma_{y,lig}$ = 1122 ± 114 MPa. Again, reference values from Berkovich tests ($\varepsilon_{i}\approx$ 7.2%) are in good agreement with spherical indentation data. Berkovich values are plotted at a total strain of 7.2%. One could argue that this value should rather refer to a plastic strain $\varepsilon_{pl}$ of 7.2% but since the initial regime of the flow curves has already a partly plastic character the evaluation of $\varepsilon_{pl}$ is disputable. Nevertheless, this would only cause a minor shift of the pyramidal indentation data and values would still be in good agreement. Only the 300 °C value deviates, the actual origin of which remains unclear at this point. No hints of different local porosities or inclusions in the vicinity of the indentation test field were noted. Potentially, enhanced diffusion at *T_hom_* of 0.43 could lead to lower stress values using sharp tips, as more pronounced stress concentrations at the edges of the pyramid promote diffusion processes [62]. Even though a hardening by annealing effect was substantiated in a previous studies [14,15,63], the effect of re-annealing, here indirectly by HT testing, was unclear so far. Figure 4b contrasts spherical indentation flow curves of RT measurements prior and after HT testing. It is evident that neither the elastic nor the plastic behavior changes significantly. Thus, foams obtained by the presented fabrication route can be considered extremely thermally stable after the initial heat treatment. The high fraction of grain-boundaries, due to the nanocrystalline microstructure, does apparently not promote the diffusion as supposed previously [63]. In addition, the formation of an oxide due to remaining Fe can be excluded as this would certainly change the mechanical properties.

**Figure 5:**
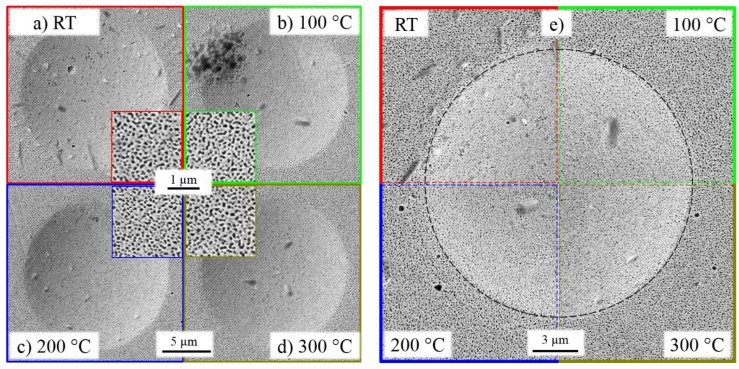
(**a**–**d**) Residual impressions of HT indentation experiments on NP Au do not show any distinctive features; (**e**) Direct comparison of indentations for each temperature condition verifies that no major drift influences or tip changes were occurring.

Figure 5a–d show residual indentations of the tested temperature conditions. Beside minor contamination of the surface no irregularities are observed. As expected for foams made of ductile materials, no pile-up or sink-in is observable as the replaced volume finds space in the open cells of the NP Au. Figure 5e displays the residual impressions of equivalent (lower right) quarters of the SEM images. Hence, no influences of non-symmetric tip imperfections have to be expected. It is clearly visible that these four images are hardly distinguishable and feature the same size. On the one hand, that means that the set displacement was reached as expected for all tests, thus potential errors from thermal drift can be excluded. In addition, the topologies of the residual imprints are alike, meaning that effects such as pile-up or sink-in are apparently not dependent on the deformation temperature. In addition, the differing work-hardening does not manifest in the impression’s morphology.

**Figure 6:**
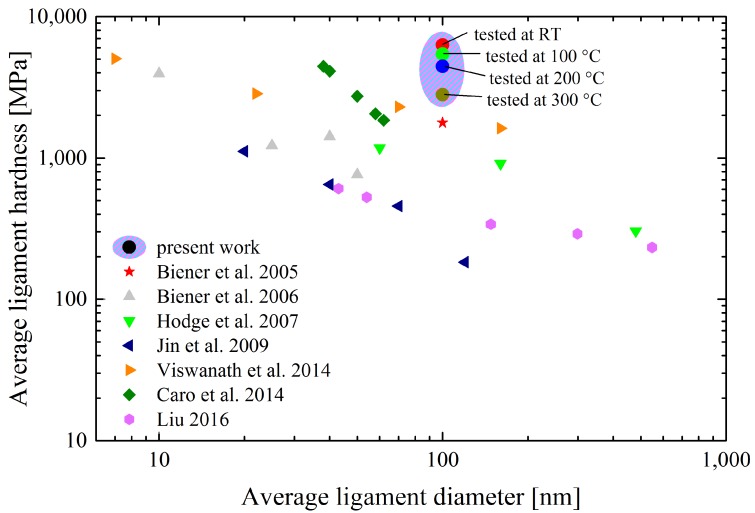
Comparison of literature ligament hardness values of NP Au foams with the present material in dependence of the ligament diameter [2,4,14,40,65,66,67].

In order to compare NP Au of different studies reasonably, Figure 6 depicts the ligament hardness *H_lig_* in dependence of the ligament diameter *d_lig_*. *H_lig_* can be calculated equivalently to Equation (1), assuming C* constant. Thus, concerns about the value of C* and varying porosities in different studies can be evaded. Data from microhardness Vickers measurements were corrected according to [64], while uniaxial data was converted to *H* by using C* = 2.5 for the strength values obtained at a strain of around 7.2%. Considering the logarithmic scale in Figure 6, it becomes unambiguous that the remarkable performance of NP Au must mainly be caused by the nanocrystalline grain structure, as it is the only distinctive feature compared to dealloyed NP Au data. Consequently, the presented fabrication route enormously contributes to a further strengthening of nanoporous structures due to grain refinement favored by mechanical alloying.

## 4. Conclusions

Implementation of spherical nanoindentation protocols is a convenient approach to assess the flow behavior of modern high-performance materials which are fabricated at a laboratory bench scale. In the present case, ultra-strong nanoporous Au was fabricated by severe plastic deformation of an AuFe precursor with subsequent selective etching. The reduction of the grain size down to the nanoscale combined with ligament diameters below 100 nm is the origin of the extraordinary high strength of the material. This was evidenced by investigations of the gradually changing microstructure in regard to the disk radius. Spherical nanoindentation experiments revealed bulk strengths of 267 ± 5 MPa in the most homogenous regions which accord to ligament strengths of more than 2.5 GPa at RT, a value close to the theoretical strength of Au [68,69]. Due to the its strong tendency to coarsen at intermediate temperatures, high-temperature data of dealloyed NP Au is generally lacking in literature [11,12,13,14]. The present study fills this gap and demonstrates that even at high temperatures up to *T_hom_* = 0.43 remarkable values of $\sigma_{y}$ (119 ± 12 MPa) and $\sigma_{y,lig}$ (1122 ± 114 MPa) are reached. Hence, the used fabrication route, involving severe plastic deformation and mechanical alloying, can certainly be considered as a potential way to further improve the performance of NP materials. Furthermore, the thorough comparison to previously reported Berkovich measurements allows to further ascertain the recently developed spherical indentation protocol.

## References

[1] Biener J., Hodge A.M., Hamza A.V. (2005). Microscopic failure behavior of nanoporous gold. Appl. Phys. Lett..

[2] Biener J., Hodge A.M., Hamza A.V., Hsiun L.M., Satcher J.H. (2005). Nanoporous Au: A high yield strength material. J. Appl. Phys..

[3] Volkert C.A., Lilleodden E.T., Kramer D., Weissmüller J. (2006). Approaching the theoretical strength in nanoporous Au. Appl. Phys. Lett..

[4] Hodge A.M., Biener J., Hayes J.R., Bythrow P.M., Volkert C.A., Hamza A.V. (2007). Scaling equation for yield strength of nanoporous open-cell foams. Acta Mater..

[5] Balk T.J., Eberl C., Sun Y., Hemker K.J., Gianola D.S. (2009). Tensile and compressive microspecimen testing of bulk nanoporous gold. JOM.

[6] Briot N.J., Kennerknecht T., Eberl C., Balk T.J. (2014). Mechanical properties of bulk single crystalline nanoporous gold investigated by millimetre-scale tension and compression testing. Philos. Mag..

[7] Mameka N., Wang K., Markmann J., Lilleodden E.T., Weissmüller J. (2016). Nanoporous Gold—Testing Macro-scale Samples to Probe Small-scale Mechanical Behavior. Mater. Res. Lett..

[8] Jin H.J., Weissmüller J. (2011). A material with electrically tunable strength and flow stress. Science.

[9] Pickering H.W. (1983). Characteristic features of alloy polarization curves. Corros. Sci..

[10] Erlebacher J., Aziz M.J., Karma A., Dimitrov N., Sieradzki K. (2001). Evolution of nanoporosity in dealloying. Nature.

[11] Seker E., Reed M.L., Begley M.R. (2009). Nanoporous gold: Fabrication, characterization, and applications. Materials (Basel).

[12] Seker E., Gaskins J.T., Bart-Smith H., Zhu J., Reed M.L., Zangari G., Kelly R., Begley M.R. (2007). The effects of post-fabrication annealing on the mechanical properties of freestanding nanoporous gold structures. Acta Mater..

[13] Hakamada M., Mabuchi M. (2007). Mechanical strength of nanoporous gold fabricated by dealloying. Scr. Mater..

[14] Viswanath R.N., Polaki S.R., Rajaraman R., Abhaya S., Chirayath V.A., Amarendra G., Sundar C.S. (2014). On the scaling behavior of hardness with ligament diameter of nanoporous-Au: Constrained motion of dislocations along the ligaments. Appl. Phys. Lett..

[15] Leitner A., Maier-Kiener V., Jeong J., Abad M.D., Hosemann P., Oh S.H., Kiener D. (2016). Interface dominated mechanical properties of ultra-fine grained and nanoporous Au at elevated temperatures. Acta Mater..

[16] Bachmaier A., Kerber M., Setman D., Pippan R. (2012). The formation of supersaturated solid solutions in Fe-Cu alloys deformed by high-pressure torsion. Acta Mater..

[17] Kormout K.S., Pippan R., Bachmaier A. (2017). Deformation-induced supersaturation in immiscible material systems during High-Pressure Torsion. Adv. Eng. Mater..

[18] Tabor D. (1951). The Hardness of Metals.

[19] Kalidindi S.R., Pathak S. (2008). Determination of the effective zero-point and the extraction of spherical nanoindentation stress—Strain curves. Acta Mater..

[20] Pathak S., Kalidindi S.R. (2015). Spherical nanoindentation stress-strain curves. Mater. Sci. Eng. R Rep..

[21] Leitner A., Maier-Kiener V., Kiener D. (2018). Essential refinements of spherical nanoindentation protocols for the reliable determination of mechanical flow curves. Mater. Des..

[22] Pippan R., Scheriau S., Hohenwarter A., Hafok M. (2008). Advantages and Limitations of HPT: A Review. Mater. Sci. Forum.

[23] Valiev R.Z., Islamgaliev R.K., Alexandrov I.V. (2000). Bulk nanostructured materials from severe plastic deformation. Prog. Mater. Sci..

[24] Huang X., Hansen N., Tsuji N. (2006). Hardening by Annealing and Softening by Deformation in Nanostructured Metals. Science.

[25] Renk O., Hohenwarter A., Eder K., Kormout K.S., Cairney J.M., Pippan R. (2015). Increasing the strength of nanocrystalline steels by annealing: Is segregation necessary?. Scr. Mater..

[26] Taylor G.I. (1934). The Mechanism of Plastic Deformation of Crystals. Part II. Comparison with Observations. Proc. R. Soc. A Math. Phys. Eng. Sci..

[27] Vorhauer A., Pippan R. (2004). On the homogeneity of deformation by high pressure torsion. Scr. Mater..

[28] Pourbaix M. (1966). Atlas of Electrochemical Equilibria in Aqueous Solutions.

[29] Kreuzeder M., Abad M.-D., Primorac M.-M., Hosemann P., Maier V., Kiener D. (2015). Fabrication and thermo-mechanical behavior of ultra-fine porous copper. J. Mater. Sci..

[30] Uchic M.D., Dimiduk D.M. (2005). A methodology to investigate size scale effects in crystalline plasticity using uniaxial compression testing. Mater. Sci. Eng. A.

[31] Sabirov I., Estrin Y., Barnett M.R., Timokhina I., Hodgson P.D. (2008). Tensile deformation of an ultrafine-grained aluminium alloy: Micro shear banding and grain boundary sliding. Acta Mater..

[32] Patel D.K., Kalidindi S.R. (2016). Correlation of spherical nanoindentation stress-strain curves to simple compression stress-strain curves for elastic-plastic isotropic materials using finite element models. Acta Mater..

[33] Prandtl L. (1920). Über die Härte plastischer Körper. Nachrichten von der Gesellschaft der Wissenschaften zu Göttingen.

[34] Atkins A.G., Tabor D. (1965). Plastic indentation in metals with cones. J. Mech. Phys. Solids.

[35] Mesarovic S.-D., Fleck N.A. (1999). Spherical indentation of elastic-plastic solids. Proc. R. Soc. Lond. A Math. Phys. Eng. Sci..

[36] Luehrs L., Soyarslan C., Markmann J., Bargmann S., Weissmüller J. (2016). Elastic and plastic Poisson’s ratios of nanoporous gold. Scr. Mater..

[37] Gibson L.J., Ashby M.F. (1997). Cellular solids: Structure and properties. Chap.

[38] Ruestes C.J., Schwen D., Millán E.N., Aparicio E., Bringa E.M. (2018). Mechanical properties of Au foams under nanoindentation. Comput. Mater. Sci..

[39] Briot N.J., Balk T.J. (2018). Focused ion beam characterization of deformation resulting from nanoindentation of nanoporous gold. MRS Commun..

[40] Jin H.-J., Kurmanaeva L., Schmauch J., Rösner H., Ivanisenko Y., Weissmüller J. (2009). Deforming nanoporous metal: Role of lattice coherency. Acta Mater..

[41] Mangipudi K.R., Epler E., Volkert C.A. (2018). On the multiaxial yielding and hardness to yield stress relation of nanoporous gold. Scr. Mater..

[42] Oliver W.C., Pharr G.M. (1992). An improved technique for determining hardness and elastic modulus using load and displacement sensing indentation experiments. J. Mater. Res..

[43] Sneddon I. (1965). The relation between load and penetration in the axisymmetric Boussinesq problem for a punch of arbitrary profile. Int. J. Eng. Sci..

[44] Kim Y.C., Gwak E.J., Ahn S.M., Jang J.I., Han H.N., Kim J.Y. (2017). Indentation size effect in nanoporous gold. Acta Mater..

[45] Kim Y.C., Gwak E.J., Ahn S.M., Kang N.R., Han H.N., Jang J.I., Kim J.Y. (2018). Indentation size effect for spherical nanoindentation on nanoporous gold. Scr. Mater..

[46] Bigl S., Schöberl T., Wurster S., Cordill M.J., Kiener D. (2016). Correlative microstructure and topography informed nanoindentation of copper films. Surf. Coat. Technol..

[47] Hall E.O. (1951). The Deformation and Ageing of Mild Steel: Discussion of Results. Proc. Phys. Soc. Sect. B.

[48] Petch N.J. (1953). The cleavage strength of polycrystals. J. Iron Steel Inst..

[49] Lee D., Wei X., Chen X., Zhao M., Jun S.C., Hone J., Herbert E.G., Oliver W.C., Kysar J.W. (2007). Microfabrication and mechanical properties of nanoporous gold at the nanoscale. Scr. Mater..

[50] Wheeler J.M., Michler J. (2013). Invited Article: Indenter materials for high temperature nanoindentation. Rev. Sci. Instrum..

[51] Wheeler J.M., Armstrong D.E.J., Heinz W., Schwaiger R. (2015). High temperature nanoindentation: The state of the art and future challenges. Curr. Opin. Solid State Mater. Sci..

[52] Durst K., Maier V. (2015). Dynamic nanoindentation testing for studying thermally activated processes from single to nanocrystalline metals. Curr. Opin. Solid State Mater. Sci..

[53] Maier V., Merle B., Göken M., Durst K. (2013). An improved long-term nanoindentation creep testing approach for studying the local deformation processes in nanocrystalline metals at room and elevated temperatures. J. Mater. Res..

[54] Hay J.L., Oliver W.C., Bolshakov A., Pharr G.M. (1998). Using the ratio of loading slope and elastic stiffness to predict pile-up and constraint factor during indentation. MRS Proc..

[55] Bolshakov A., Pharr G.M. (1998). Influences of pileup on the measurement of mechanical properties by load and depth sensing indentation techniques. J. Mater. Res..

[56] Randall N.X., Julia-Schmutz C. (1998). Evalution of contact area and pile-up during the nanoindentation of soft coatings on hard substrates. Mat. Res. Soc. Symp. Proc..

[57] Maier V., Leitner A., Pippan R., Kiener D. (2015). Thermally Activated Deformation Behavior of ufg-Au: Environmental Issues during Long-Term and High-Temperature Nanoindentation Testing. JOM.

[58] Hodge A.M., Doucette R.T., Biener M.M., Biener J., Cervantes O., Hamza A.V. (2009). Ag effects on the elastic modulus values of nanoporous Au foams. J. Mater. Res..

[59] Soyarslan C., Bargmann S., Pradas M., Weissmüller J. (2018). 3D stochastic bicontinuous microstructures: Generation, topology and elasticity. Acta Mater..

[60] Hu K., Ziehmer M., Wang K., Lilleodden E.T. (2016). Nanoporous gold: 3D structural analyses of representative volumes and their implications on scaling relations of mechanical behaviour. Philos. Mag..

[61] Huber N., Viswanath R.N., Mameka N., Markmann J., Weißmüller J. (2014). Scaling laws of nanoporous metals under uniaxial compression. Acta Mater..

[62] Gangulee A. (1970). Stress-enhanced diffusion in thin films. Philos. Mag..

[63] Koifman Khristosov M., Dishon S., Noi I., Katsman A., Pokroy B. (2017). Pore and Ligament Size Control, Thermal Stability and Mechanical Properties of Nanoporous Single Crystals of Gold. Nanoscale.

[64] Leitner A., Maier-Kiener V., Kiener D. (2017). Extraction of Flow Behavior and Hall–Petch Parameters Using a Nanoindentation Multiple Sharp Tip Approach. Adv. Eng. Mater..

[65] Biener J., Hodge A.M.A., Hayes J.R.J., Volkert C.A., Zepeda-Ruiz L.A., Hamza A.V., Abraham F.F. (2006). Size effects on the mechanical behavior of nanoporous Au. Nano Lett..

[66] Caro M., Mook W.M., Fu E.G., Wang Y.Q., Sheehan C., Martinez E., Baldwin J.K., Caro A. (2014). Radiation induced effects on mechanical properties of nanoporous gold foams. Appl. Phys. Lett..

[67] Liu L.Z., Ye X.L., Jin H.J. (2016). Interpreting anomalous low-strength and low-stiffness of nanoporous gold: Quantification of network connectivity. Acta Mater..

[68] Kiely J.D., Houston J.E. (1998). Nanomechanical properties of Au (111), (001), and (110) surfaces. Phys. Rev. B.

[69] Stalder A., Dürig U. (1996). Study of yielding mechanics in nanometer-sized Au contacts. Appl. Phys. Lett..

